# Enforced monoandry over generations induces a reduction of female investment into reproduction in a promiscuous bird

**DOI:** 10.1111/eva.13311

**Published:** 2021-11-13

**Authors:** Gabriele Sorci, Loïc Lesobre, Pauline Vuarin, Gwènaëlle Levêque, Michel Saint Jalme, Frédéric Lacroix, Yves Hingrat

**Affiliations:** ^1^ Biogéosciences UMR 6282 CNRS Université de Bourgogne Franche‐Comté Dijon France; ^2^ Reneco International Wildlife Consultants LLC Abu Dhabi United Arab Emirates; ^3^ 194913 Emirates Center for Wildlife Propagation Missour Morocco; ^4^ 52827 Centre d’Ecologie et des Sciences de la Conservation UMR 7204 MNHN CNRS‐UPMC Museum National d'Histoire Naturelle Paris France; ^5^ Present address: 27098 Laboratoire de Biométrie et Biologie Evolutive ‐ UMR CNRS 5558 Université Claude Bernard Lyon 1 16 rue Raphaël Dubois 69622 Villeurbanne Cedex France

**Keywords:** ex situ conservation, female multiple mating, polyandry, postcopulatory sexual selection, reproductive investment

## Abstract

While uncovering the costs and benefits of polyandry has attracted considerable attention, assessing the net effect of sexual selection on population fitness requires the experimental manipulation of female mating over generations, which is usually only achievable in laboratory populations of arthropods. However, knowing if sexual selection improves or impairs the expression of life‐history traits is key for the management of captive populations of endangered species, which are mostly long‐lived birds and mammals. It might therefore be questionable to extrapolate the results gathered on laboratory populations of insects to infer the net effect of sexual selection on populations of endangered species. Here, we used a longitudinal dataset that has been collected on a long‐lived bird, the houbara bustard, kept in a conservation breeding program, to investigate the effect of enforced monoandry on female investment into reproduction. In captivity, female houbara bustards are artificially inseminated with sperm collected from a single male (enforced monoandry), or sequentially inseminated with semen of different males (polyandry), allowing postcopulatory sexual selection to operate. We identified female lines that were produced either by monoandrous or polyandrous inseminations over three generations, and we compared reproductive investment of females from the two mating system groups. We found that females in the polyandrous lines had higher investment into reproduction as they laid more eggs per season and produced heavier hatchlings. Higher reproductive investment into reproduction in the polyandrous lines did not result from inherited differences from females initially included in the two mating system groups. These results show that removal of sexual selection can alter reproductive investment after only few generations, potentially hindering population fitness and the success of conservation breeding programs.

## 1 INTRODUCTION

Polyandry, the mating system where females mate with more than one male during a single reproductive event, is widespread among animals. Nonetheless, extensive among‐species and among‐population variation in the proportion of polyandrous females exists (Brouwer & Griffith, 2019; Taylor et al., 2014). While female multiple mating might have evolved for both adaptive and nonadaptive reasons (Boulton et al., 2018; Forstmeier et al., 2014; Holman, 2015; Kokko & Mappes, 2012), it is often assumed that polyandrous females gather direct and/or indirect benefits by mating with multiple males (Evans, 2012; Martin et al., 2004; Thonhauser et al., 2013). Benefits range from direct acquisition of resources, that improve fertility and fecundity, to genetic (additive or compatibility) effects, possibly improving offspring fitness (Barbosa et al., 2012; Croshaw et al., 2017; Evans, & Magurran, 2000; Fisher et al., 2006; Gerlach et al., 2012; Gowaty et al., 2010; Head et al., 2005; Newcomer et al., 1999; Slatyer et al., 2012; Taylor et al., 2008). Evidence supporting the view that multiple mating improves female fitness is, however, mixed and several studies have failed to report any benefit of polyandry (Arbuthnott, & Rundle, 2012; Chenoweth et al., 2015; Holland, 2002; Lumley et al., 2016). For instance, a recent study showed that isofemale lines of *Drosophila pseudoobscura* with different levels of polyandry had similar fitness, and the frequency of polyandry remained stable over generations, further suggesting no selective advantage for highly polyandrous genotypes (Sutter et al., 2019).

Multiple mating is not cost‐free for females, since they might be exposed to harmful male harassment, the transfer of toxic compounds or sexually transmitted diseases through male semen, potentially reducing female fecundity and life span (Chapman et al., 1995; Crudgington, & Siva‐Jothy, 2000; Lew et al., 2006; Wigby, & Chapman, 2005). Furthermore, female multiple mating extends the opportunity for sexual selection to operate on males through sperm competition and/or cryptic female choice (Birkhead, & Pizzari, 2002; Simmons, 2005). Such postcopulatory sexual selection has been shown to drive the evolution of several male traits improving the likelihood of egg fertilization when ejaculates of several males compete in the female reproductive tract (Fitzpatrick, & Lüpold, 2014; Parker, & Pizzari, 2010; Simmons, & Fitzpatrick, 2012). However, traits that improve male reproductive success during postcopulatory sexual selection might be negatively correlated with female reproductive success giving rise to sexual conflict (Edward et al., 2010; Parker, 2006; Pischedda, & Chippindale, 2006). Therefore, the finding of costs of multiple mating and of sexual conflict questions the net effect of sexual selection on population fitness.

In that respect, it has been shown that allowing sexual selection to operate might improve population fitness, allowing populations to better face environmental hazards (Price et al., 2010). For instance, lines of the flour beetle (*Tribolium castaneum*) evolving under strong sexual selection over many generations were more likely to persist when facing high inbreeding level compared to lines evolving under weak sexual selection (Lumley et al., 2015). Applying other environmental stresses over a three‐generation cycle (nutritional stress, thermal stress, and genetic bottleneck, one stress type per generation) to flour beetles having evolved under a polyandrous mating system confirmed a higher resistance to extinction compared to individuals having evolved under enforced monogamy (Godwin et al., 2020). The link between sexual selection and the persistence of populations experiencing deteriorated environments has recently also been shown under natural conditions (Parrett et al., 2019).

In addition to its fundamental interest, understanding how sexual selection affects population fitness also has far‐reaching consequences for the conservation of endangered species, especially in terms of captive population management (Ashley et al., 2003; Chargé et al., 2014; Farquharson et al., 2020; Holman & Kokko, 2013; Martinez‐Ruiz, & Knell, 2017; Schulte‐Hostedde, & Mastromonaco, 2015; Wedekind, 2002). Ex situ conservation programs aim to maintain and reproduce endangered species in captivity to restore declining natural populations (Ebenhard, 1995). However, in captivity, pairing usually occurs according to a genetic management strategy, aiming at maintaining genetic diversity, which leaves little place for sexual selection to operate. Recent findings have, however, questioned the relevance of a full removal of sexual selection in captive breeding (Martin‐Wintle et al., 2018), since allowing females (and males) to choose a partner might improve reproductive success, as shown, for instance, for the iconic giant panda (*Ailuropoda melanoleuca*) (Martin‐Wintle et al., 2015). However, on the negative side, allowing sexual selection to operate, might result in a small number of males contributing to the next generation (reproductive skew), with an associated loss of genetic diversity. For instance, male reproduction of the endangered Tasmanian devil (*Sarcophilus harrisii*) was highly skewed (60% of males not contributing to the next generation) when individuals were housed in groups and allowed to mate freely (Farquharson et al., 2019). The effect of sexual selection on long‐term population fitness and genetic diversity in captive endangered species has yet to be investigated.

Experimental evolution is certainly the most relevant approach to infer the evolutionary consequences of sexual selection on fitness‐linked traits (Demont et al., 2014; Edward et al., 2010). Usually, these studies remove sexual selection by enforcing single male mating, whereas other experimental lines are allowed to mate freely (allowing polyandry); in some cases, a more quantitative approach is used where the sex ratio of the population is manipulated (usually the number of males), altering the strength of the sexual selection (Nandy et al., 2013). However, it should be noted that the experimental evolution approach is restricted to species that can be maintained in the laboratory and have short generation time, therefore essentially invertebrates, notable exceptions being the house mouse (*Mus domesticus*) and the guppy (*Poecilia reticulata*) (Firman, & Simmons, 2012; Pélabon et al., 2014), but it is not clear whether these results may be extended to other organisms with different life‐history traits (e.g., large‐bodied, long‐lived species). Given that the vast majority of endangered species kept *ex situ* are vertebrates (mostly, birds and mammals), it might prove difficult to extrapolate results from insects to inform stakeholders on how best to implement sexual selection into the management of captive populations. Moreover, allowing females to freely mate with one or several males does indeed manipulate the opportunity for sexual selection to occur, but usually does not control for the number of matings or the actual number of different partners of a female, possibly confounding direct effects on fecundity and indirect (genetic) ones. Artificial inseminations (AI) and in vitro fertilizations (IVF) are alternative approaches allowing to control these potential confounding effects. However, running experimental evolution using AI or IVF further reduces the spectrum of candidate organisms where such experiments can be conducted.

Here, we took advantage of a unique longitudinal dataset on a nondomesticated bird with a promiscuous mating system (the North African houbara bustard, *Chlamydotis undulata*) (Hingrat et al., 2007; Lesobre, Lacroix, Le Nuz, et al., 2010), maintained in a conservation breeding program (Rabier et al., 2020) to investigate the effect of enforced monoandry on female reproductive investment. In this program, females and males are kept in isolation and breeding only occurs through AI. Therefore, no precopulatory mate choice can occur. However, depending on the daily availability of semen and the genetic management of the flock (Lesobre, Lacroix, Caizergues, et al., 2010; Rabier et al., 2020), females can be inseminated with single male sperm or sequentially with sperm from different males. When females are inseminated with sperm from a single male, no sexual selection can occur at all (neither pre‐ nor postcopulatory selection). Nevertheless, when females are inseminated with sperm from different males, sperm competition and cryptic female choice are potentially at play (Vuarin, Bouchard, et al., 2019; Vuarin, Hingrat, et al., 2019). The houbara bustard is a long‐lived species, and given that the program has run for more than 20 years, we were able to retrospectively identify lines of females that were consistently produced through single male (enforced monoandry) or multimale inseminations (polyandry), over three generations.

Comparing monoandrous and polyandrous female lines, we tested whether enforced monoandry affected female investment into four reproductive traits: date of first egg laid, number of eggs laid over the season, egg mass, and hatchling mass. Given that female houbara bustards express a high frequency of polyandry in nature, as indicated by clutches with multiple paternity (Lesobre, Lacroix, Le Nuz, et al., 2010), we tentatively predicted that enforced monoandry over generations might negatively affect female fitness in this species.

## 2 MATERIAL AND METHODS

Birds used in this study are part of the Emirates Center for Wildlife Propagation (ECWP), a conservation breeding aiming at reinforcing natural populations of the North African houbara bustard, which entirely relies on AI (Saint Jalme et al., 1994). When females are deemed to be ready to lay, they are inseminated with fresh (daily collected) sperm. Before the first egg of the clutch is laid, two sequential inseminations are performed with a 48‐h interval (but occasionally females can lay after the first insemination); a third and fourth (and so on) insemination can be added if required (i.e., if the first egg of the clutch takes longer to come than expected). Therefore, depending on when the egg is laid, females can be inseminated once, twice, etc. Such successive inseminations can involve sperm collected from the same male or from different males [with each insemination involving sperm from a single male (e.g., no mix of sperm was used)]. When females are successively inseminated with sperm from different males, postcopulatory sexual selection can occur. Clutch size varies between 2 and 3 eggs. In nature, female houbara bustards lay one clutch per season. However, if the clutch is lost (e.g., due to predation) females lay replacement clutches. In the captive breeding, on the day of laying, all eggs are collected and brought to the incubation facility where they are weighted and incubated. Egg collection, therefore, stimulates females to lay replacement clutches. At hatching, chicks are weighed and then raised following specific protocols, depending on whether birds are deemed to be released in the field or kept as future breeders.

Using the database with all the records of sperm collection events and female inseminations, we retrospectively assessed whether females were produced following single or multimale sperm inseminations, allowing us to identity lines of females that experienced a full removal of sexual selection or lines where postcopulatory sexual selection was allowed to operate. Given that females can store sperm in dedicated structures for weeks, we adopted a very conservative rule to assign females to either monoandrous or polyandrous lines. For each egg, we identified all the inseminations that occurred in the 40 days that preceded egg laying. If all of them involved sperm collected from the same male, the egg was considered as being eligible for inclusion in a monoandrous line, if sperm from different males was used to inseminate the female during the 40 days preceding laying, the egg was considered eligible for inclusion in a polyandrous line. The 40‐day threshold was based on the finding that inseminations occurring more than 40 days prior to egg laying never result in successful fertilization (unpublished data). We were able to reconstruct lines that covered up to three generations, spanning the period 2004–2019 (52 monoandrous and 35 polyandrous lines). A monoandrous line was defined as a female that (1) was produced following a single male insemination of her mother; (2) whose mother was produced following a single male insemination of her grandmother; (3) whose grandmother was produced following a single male insemination of her great grandmother. A polyandrous line was defined as a female that (1) was produced following a multimale insemination of her mother; (2) whose mother was produced following a multimale insemination of her grandmother; (3) whose grandmother was produced following a multimale insemination of her great grandmother. On average, females in the polyandrous lines were produced from inseminations involving sperm from 3.41 males over the three generations (min = 2, max = 8). Note that all third‐generation females produced by both monoandrous and polyandrous lines (the focal females of the study) were inseminated with single and multimale inseminations during their lifetime. Figures S1 and S2 illustrate the rationale used to assign females to either a monoandrous or a polyandrous line, and the insemination scheme of the third‐generation focal females.

We used four traits describing the reproductive investment of females in the monoandrous and polyandrous lines: date of first egg laid per season, total number of eggs laid per season, egg mass, and hatchling mass.

### 2.1 Statistical analyses

To assess the effect of mating system on traits describing female investment into reproduction, we used linear mixed effects models (LMM). Models on date at first egg laid, egg mass, and hatchling mass had a normal distribution of errors, whereas the model on number of eggs laid per season had a Poisson distribution of errors. Each model had a similar structure, with line (equivalent to female ID), year of birth, and year of data collection included as random factors (the model on number of eggs laid only included line and year of birth because the inclusion of the third random factor prevented the model from converging). Each model also included several fixed effects that differed according to the response variable. The model on the date of first egg laid was the simplest one since it only included the mating system (monoandry vs. polyandry), female age (log‐transformed), squared age, and the two‐way interactions between mating system and age. Owing to small sample size, females older than 10 years were grouped in the same age class. The model on the number of eggs laid, in addition to the mating system and age (both linear and quadratic terms), also included the date of first egg laid, the maximum number of inseminations received per breeding season, the maximum number of males contributing to the inseminations per breeding season, and the two‐way interactions between mating system and each covariate. Models on egg and hatchling mass included the following fixed effects: mating system (monoandry vs. polyandry), age (both linear and quadratic), egg position in the laying sequence, date of egg laying (linear and quadratic), number of males contributing to the inseminations, and the two‐way interactions between mating system and each covariate. Covariates were standardized with mean = 0 and *SD* = 1 to facilitate comparison of parameter estimates. Models were selected using a backward procedure. The final models are reported in the result section, while the initial models are reported in the online supplemental material.

All LMMs were run using PROC MIXED (for response variables with normal error distribution) and PROC GLIMMIX (for the response variable with Poisson error distribution) (SAS).

## 3 RESULTS

### 3.1 Laying date

Monoandrous female lines started to lay their eggs at a similar date compared to polyandrous female lines (Table 1 and Table S1). Only age was correlated with date of first egg laid, with females starting to lay at earlier dates as they became older (Table 1 and Table S1).

**TABLE 1:** Linear mixed effects model exploring the effect of mating system on date of first egg laid

*Fixed effects*	*Parameter estimate*	*SE*	*t*	*p*	*95% CI*
Intercept (monoandry)	83.96	3.95			
Mating system (polyandry)	1.03	3.70	0.28	0.7818	−6.26/8.31
Age	−5.39	1.0.3	−5.21	<0.0001	−7.42/−3.35

*Random effects*	*Estimate*	*SE*	*z*	*p*
Line	195.14	42.62	4.58	<0.0001
Year of birth	93.51	69.82	1.34	0.0902
Year of data collection	15.02	11.30	1.33	0.0919
Residual	180.94	14.39	12.57	<0.0001

Note

The model included the mating system (monoandry or polyandry), age (log‐transformed), squared age, and the two‐way interactions between mating system and age. Line, year of birth and year of data collection were included as random effects. The analysis was based on 421 observations collected over 13 years on 87 female lines spanning 13 cohorts. The monoandrous group was set as the reference. We report parameter estimates (with SE and 95% CI), *t* and *p* values for the minimal adequate model (see online supplemental material for the initial model).

### 3.2 Number of eggs laid

We first checked whether there was a difference in the number of eggs laid by great grandmothers included in the monoandrous and polyandrous lines [*n* = 47 females (27 monoandrous and 20 polyandrous), and 92 observations]. The results showed that the number of eggs laid was similar for the females that started the monoandrous and the polyandrous groups (LS‐Means ± *SE* = 4.99 ± 0.39 and 4.87 ± 0.42 for the monoandrous and the polyandrous groups, respectively; β ± *SE* = −0.025 ± 0.112, *t* = −0.23, *p* = 0.8226).

**FIGURE 1:**
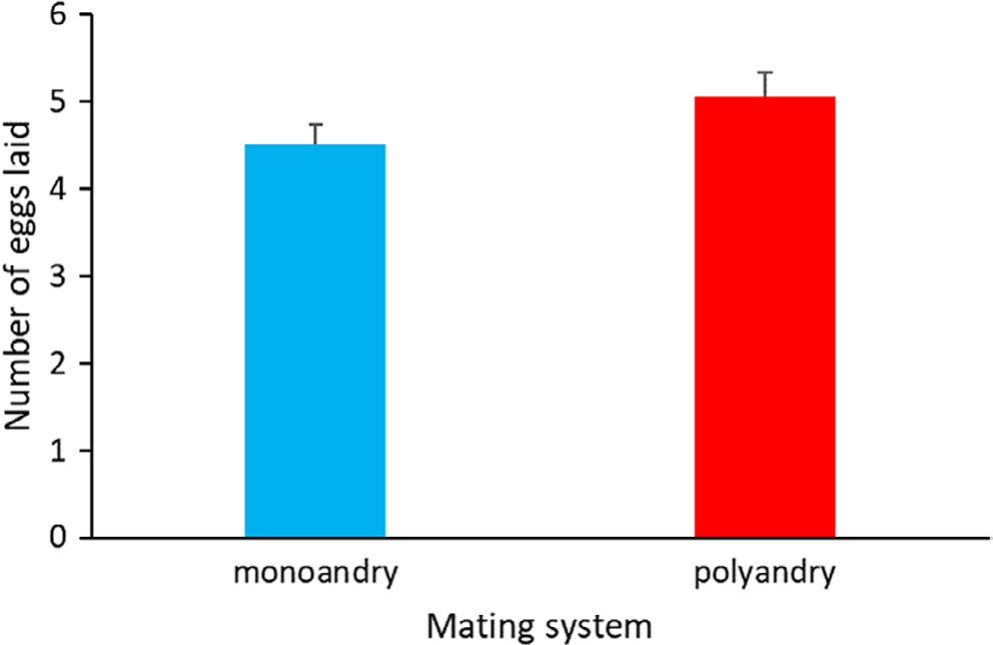
Number of eggs laid per season by female houbara bustards produced after three generations of monogamous or polyandrous matings. We report least‐squares means ±*SE*

However, after three generations of monoandrous or polyandrous inseminations, females differed in the number of eggs laid per season, with females from the polyandrous lines laying on average 10% more eggs per season (Table 2, Figure 1, Table S2). This result holds true when controlling for a number of potential confounding factors, such as the date of the first egg laid, number of inseminations, and number of males contributing to inseminations (Table 2 and Table S2). Moreover, third‐generation females from the monoandrous and polyandrous lines experienced similar number of inseminations and males (number of inseminations, β ± *SE* = −0.052 ± 0.137, *t* = −0.38, *p* = 0.7014; number of males, β ± *SE* = 0.050 ± 0.130, *t* = 0.39, *p* = 0.6998), further suggesting that the higher fecundity of polyandrous line females was not due to these potential confounding effects.

**TABLE 2:** Linear mixed effects model exploring the effect of the mating system on the number of eggs laid per season

*Fixed effects*	*Parameter estimate*	*SE*	*t*	*p*	*95% CI*
Intercept (monoandry)	1.51	0.05			
Mating system (polyandry)	0.11	0.05	2.35	0.0190	0.02/0.21
Age	0.25	0.08	2.95	0.0034	0.08/0.41
Age²	−0.30	0.08	−3.74	0.0002	−0.45/−0.14
Maximum number of inseminations	0.46	0.03	16.16	<0.0001	0.40/0.51
Maximum number of males	0.19	0.02	8.39	<0.0001	0.15/0.24
Date of first egg laid	−0.16	0.02	−7.84	<0.0001	−0.20/−0.12

*Random effects*	*Estimate*	*SE*
Line	0.01	0.006
Year of birth	0.015	0.014

Note

The model (which had a Poisson distribution of errors) included the mating system (monoandry or polyandry), age (log‐transformed), squared age, maximum number of inseminations, maximum number of males, date of first egg laid, and the two‐way interactions between mating system and covariates. Line and year of birth were included as random effects. The analysis was based on 609 observations collected over 13 years on 87 female lines spanning 13 cohorts. The monoandrous group was set as the reference. We report parameter estimates (with SE and 95% CI), t and p values for the minimal adequate model (see the online supplemental material for the initial model).

### 3.3 Egg and hatchling mass

As for the number of eggs laid, we first investigated if there was any difference in egg mass and hatchling mass between great grandmothers that were used to start the monoandrous and polyandrous lines. Egg mass did not differ between females initially included in the monoandrous and polyandrous groups [LS‐Means ± *SE* (g) = 60.78 ± 1.64 and 59.50 ± 1.75 for the monoandrous and the polyandrous groups, respectively; β ± *SE* = −1.284 ± 1.934, *t* = −0.66, *p* = 0.5074; *n* = 41 females and 293 observations]. Similarly, there was no difference in hatchling mass between monoandrous and polyandrous great grandmothers [LS‐Means ± *SE* (g) = 39.37 ± 1.01 and 39.95 ± 1.14 for the monoandrous and the polyandrous groups, respectively; β ± *SE* = 0.585 ± 1.342, *t* = 0.44, *p* = 0.6634; *n* = 47 females and 417 observations].

**FIGURE 2:**
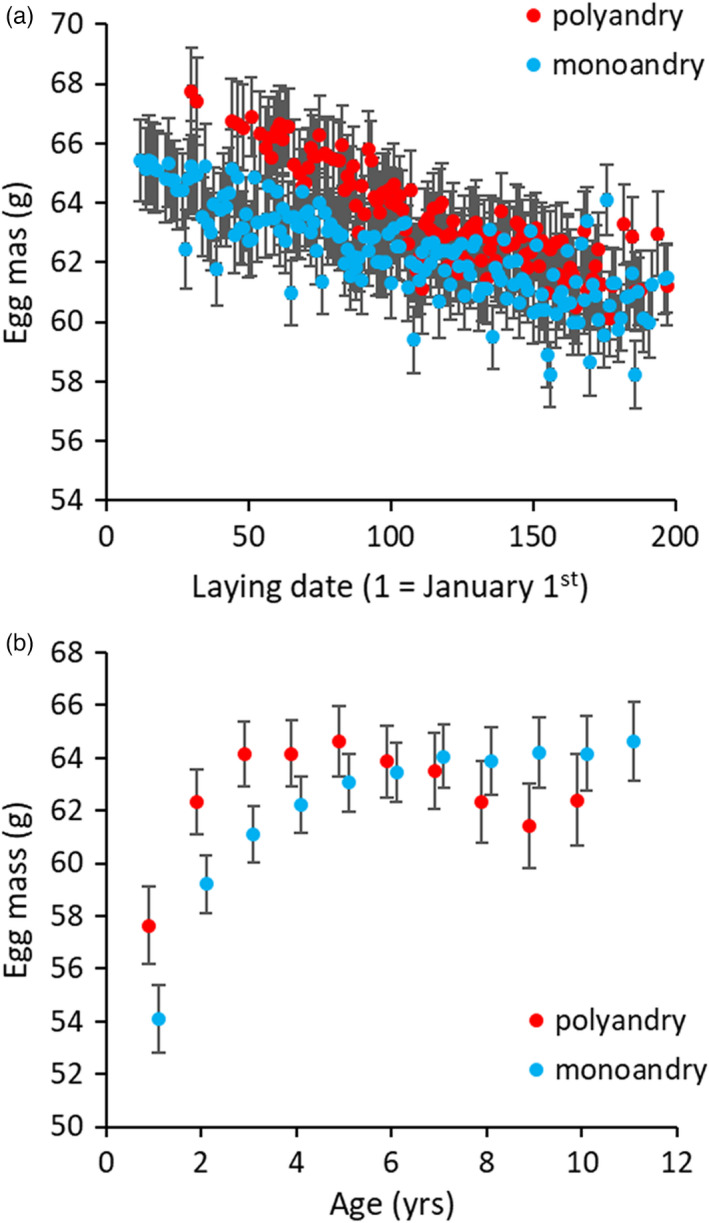
Egg mass (g) as a function of laying date (a) and female age (b) for the two mating systems (monoandry = blue dots; polyandry = red dots). Dots represent model predicted values (±*SE*). See text for details on model construction and the online supplemental material for a plot of the raw data

**FIGURE 3:**
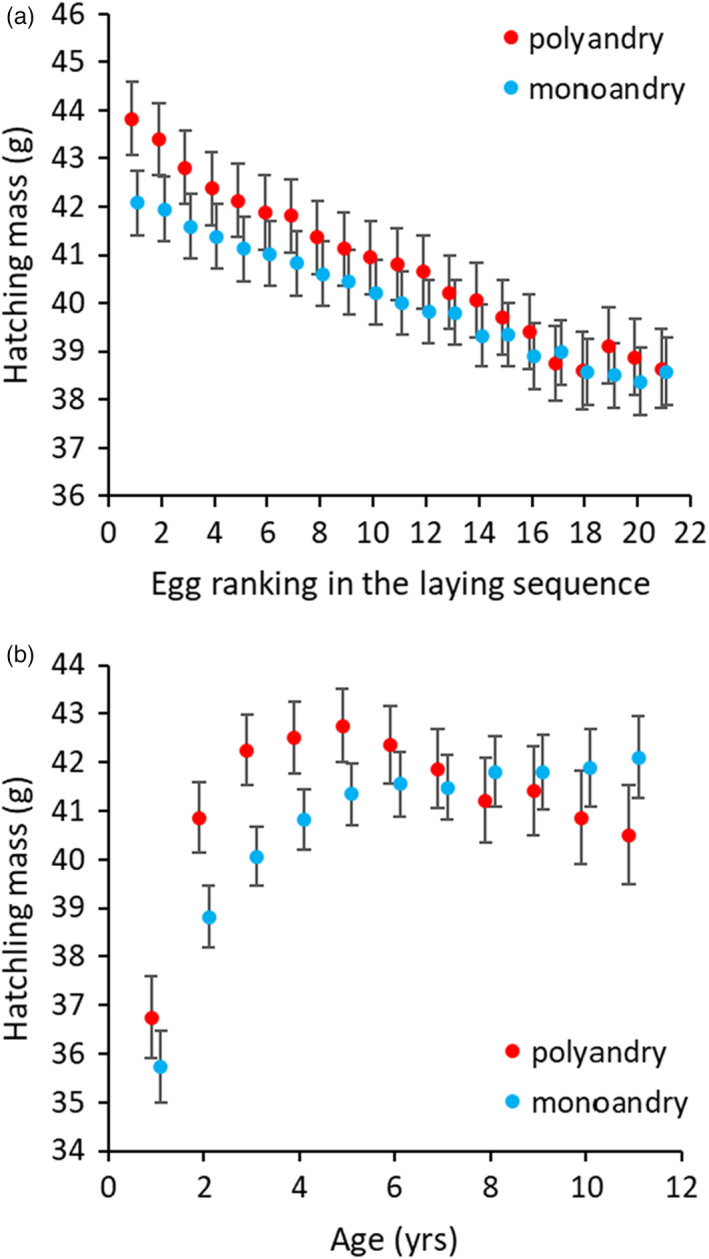
Hatchling mass (g) as a function of egg ranking in the laying sequence (a) and female age (b) for the two mating systems (monoandry = blue dots; polyandry = red dots). Dots represent model predicted values (±*SE*). See text for details on model construction and the online supplemental material for a plot of the raw data

After three generations of enforced monoandry, we found that egg size was affected by the mating system in interaction with laying date and age (Table 3 and Table S3). In particular, females from the polyandrous lines laid bigger eggs earlier during the breeding season, while the difference between the two mating systems vanished at the end of the season (Figure 2a and Figure S3). Females from the polyandrous lines also invested more into egg mass at younger ages, while females from monoandrous lines laid smaller eggs when young and showed no sign of decline in egg mass as they aged (Figure 2b and Figure S3). Females from polyandrous lines also produced heavier hatchlings at young ages and when eggs were laid earlier in the laying sequence (Table 4, Figure 3a,b; Table S4 and Figure S4).

**TABLE 3:** Linear mixed effects model exploring the effect of the mating system on egg mass (g)

*Fixed effects*	*Parameter estimate*	*SE*	*t*	*p*	*95% CI*
Intercept (monoandry)	62.06	1.05			
Mating system (polyandry)	1.50	1.35	1.11	0.2672	−1.15/4.16
Age	5.03	0.61	8.28	<0.0001	3.84/6.22
Age2	−3.12	0.73	−4.26	<0.0001	−4.56/−1.68
Egg position	−1.42	0.22	−6.41	<0.0001	−1.85/−0.98
Laying date	−0.70	0.53	−1.33	0.1823	−1.73/0.33
Laying date^2^	1.32	0.52	2.52	0.0117	0.29/2.35
Mating system (polyandry) x age	0.69	0.94	0.74	0.4610	−1.15/2.54
Mating system (polyandry) x age^2^	−2.43	0.97	−2.49	0.0128	−4.33/−0.52
Mating system (polyandry) x laying date	−0.93	0.24	−3.83	0.0001	−1.40/−0.45

*Random effects*	*Estimate*	*SE*	*z*	*p*
Line	29.15	5.28	5.52	<0.0001
Year of birth	0			
Year of data collection	3.71	1.87	1.98	0.0237
Residual	9.97	0.41	24.61	<0.0001

Note

The model included the mating system (monoandry or polyandry), age (log‐transformed), squared age, the egg position in the laying sequence, the number of males that contributed to the inseminations, laying date (linear and quadratic) and the two‐way interactions between mating system and the covariates. Line, year of birth, and year of data collection were included as random effects. The analysis was based on 1,304 observations collected over 11 years on 74 female lines spanning 12 cohorts. The monoandrous group was set as the reference. We report parameter estimates (with SE and 95% CI), t and p values for the minimal adequate model (see the online supplemental material for the initial model).

**TABLE 4:** Linear mixed effects model exploring the effect of the mating system on hatchling mass (g)

*Fixed effects*	*Parameter estimate*	*SE*	*t*	*p*	*95% CI*
Intercept (monoandry)	40.79	0.62			
Mating system (polyandry)	1.05	0.81	1.30	0.1941	−0.53/2.63
Age	3.15	0.33	9.58	<0.0001	2.51/3.80
Age^2^	−2.00	0.39	−5.13	<0.0001	−2.76/−1.23
Egg position	−1.09	0.11	−9.64	<0.0001	−1.32/−0.87
Number of males	−0.24	0.07	−3.36	0.0008	−0.38/−0.10
Laying date	−0.85	0.28	−3.03	0.0024	−1.40/−0.30
Laying date^2^	1.19	0.28	4.23	<0.0001	0.64/1.74
Mating system (polyandry) x age	1.50	0.51	2.94	0.0033	0.50/2.50
Mating system (polyandry) x age^2^	−2.48	0.54	−4.57	<0.0001	−3.54/−1.42
Mating system (polyandry) x egg position	−0.46	0.12	−3.90	<0.0001	−0.70/−0.23

*Random effects*	*Estimate*	*SE*	*z*	*p*
Line	12.52	2.04	6.14	<0.0001
Year of birth	0			
Year of data collection	1.27	0.65	1.95	0.0254
Residual	6.68	0.19	36.11	<0.0001

Note

The model included the mating system (monoandry or polyandry), age (log‐transformed), squared age, the egg position in the laying sequence, the number of males that contributed to the inseminations, laying date (linear and quadratic) and the two‐way interactions between mating system and the covariates. Line, year of birth, and year of data collection were included as random effects. The analysis was based on 2,718 observations collected over 13 years on 86 female lines spanning 13 cohorts. The monoandrous group was set as the reference. We report parameter estimates (with SE and 95% CI), *t* and *p* values for the minimal adequate model (see the online supplemental material for the initial model).

## 4 DISCUSSION

To the best of our knowledge, this is the first study showing the effect of enforced monoandry on female reproductive investment in a long‐lived bird species maintained in a conservation breeding program, while using AI to control for potential direct (fecundity‐enhancing) effects. We found that after three generations of enforced monoandry, females of the North African houbara bustard had a lowered reproductive investment in terms of number of eggs laid, egg mass, and hatchling mass, compared to females from polyandrous lines.

Experimental evolution provides the ideal tool to assess the net effect of sexual selection on fitness‐related traits, accounting for both costs and benefits associated with a specific mating system. Experimental evolution has been conducted in several organisms and a recent meta‐analysis has shown that, overall, individuals in experimental lines where sexual selection was allowed have higher values of fitness‐linked traits (Cally et al., 2019). However, the vast majority of the studies included in this meta‐analysis referred to insects and other short‐lived invertebrates. It should be fully acknowledged that the approach used in our study differs with many respects from a typical experimental evolution because we only retrospectively assigned birds to monoandrous and polyandrous lines, based on the pedigree and insemination records of the breeding flock. Nevertheless, our results add to previous evidence suggesting that full removal of sexual selection can rapidly alter the expression of key reproductive traits in a large‐bodied, long‐lived bird species.

Given that the number of eggs laid is a heritable trait in the houbara bustard (Chargé et al., 2013), the observed findings might have resulted from an initial difference in the fecundity of females included in the monoandrous and polyandrous groups. However, this is unlikely as we showed that none of the reproductive investment traits (number of eggs laid, egg mass, and hatchling mass) differed between great grandmothers included in the monoandrous and polyandrous lines. That said, we cannot precisely identify the mechanism underlying the observed changes in life‐history traits. Inseminating females with sperm of several males allows sperm competition to operate and might result in males with the best ejaculate attributes to outcompete rivals (Vuarin, Hingrat, et al., 2019). This might result in positive selection for sperm number, and previous work conducted in the houbara bustard has shown that sperm number in the ejaculate is a heritable trait (Chargé et al., 2013). Interestingly, this previous work also showed that sperm and egg number were positively genetically correlated (Chargé et al., 2013). Therefore, it is possible that the observed changes in female reproductive investment in polyandrous lines results from a correlated response to selection acting on male ejaculate attributes. In addition to this, females inseminated with semen of several males might express a postcopulatory cryptic choice for sperm from high‐quality or compatible males (Vuarin, Bouchard, et al., 2019), resulting in improved reproductive performance of the progeny.

The changes in reproductive investment occurred after only three generations of enforced monoandry. As mentioned above, such rapid changes might take place if there is a between‐sex genetic correlation in investment into reproductive traits. However, we cannot exclude that epigenetic changes might also participate to explain the observed results. Nongenetic effects on female reproductive investment (including fecundity) as a function of male attractiveness have been reported in birds (e.g., Gilbert et al., 2012). Although in our experimental setup, female houbara bustards could not assess male quality through direct visual cues, one might speculate that females might still evaluate mate quality through cues delivered with the ejaculate. Further work is definitely needed to identify the mechanisms underlying the association between enforced monoandry and reproductive investment over generations.

Although our approach allowed us to assess the net effect of polyandry on female reproductive investment, we fully acknowledge that the environmental conditions experienced by houbara bustards in the captive breeding do not reproduce those encountered in the wild. As such, several potential costs associated with multiple mating are offset in the captive breeding. These include, among others, the cost for searching mates, male harassment, or the transfer of sexually transmitted pathogens. Similarly, while using AI allows to control for several potential confounding factors, we acknowledge that females expressing a precopulatory mate choice in the wild (having a behavioral control on their multiple mating) might have different paternity bias compared to females receiving arbitrary inseminations [see for instance Gasparini and Evans (2018)].

In our study, we could assess both transgenerational and direct fecundity‐enhancing effects. Indeed, third‐generation females from the two mating system groups experienced both monoandrous and polyandrous inseminations during their lifetime. In addition to the transgenerational effects, we found that females receiving more inseminations (and from several males) laid more eggs suggesting a possible direct benefit of multiple mating. However, such a correlation cannot be granted as evidence for a causal link between number of inseminations or number of males and fecundity, because females with the highest fecundities might merely receive more inseminations (and therefore sperm from different males) over the breeding season. Therefore, inferring any direct benefit of multiple mating in this system would require further experimental investigation. Importantly, however, third‐generation females produced by monoandrous or polyandrous lines experienced similar number of inseminations and number of males per season, suggesting that the difference in annual fecundity between the two lines was not due to females from polyandrous lines receiving more inseminations from more males compared to females from monoandrous lines.

The effect of mating system on egg mass and hatchling mass was in interaction with other female traits. Females in the polyandrous lines laid bigger eggs and produced heavier hatchlings early in the breeding season and at the beginning of the laying sequence. Eggs laid late in the season or at the end of the laying sequence usually produce offspring with lower survival prospect and therefore an extra investment into these eggs might be worthless. Alternatively, given that females prepare their energetic reserves during winter and hardly replenish them during the breeding season, it could be that the exhaustion of the energetic reserves that intervenes as the breeding season progresses overrides any possible effect of polyandry. Interestingly, females from the polyandrous lines also produced bigger eggs and hatchlings at younger ages compared to the monoandrous lines. This finding suggests a possible age‐dependent adjustment of reproductive investment according to the mating system, with polyandrous lines investing more at early ages and showing an earlier onset and/or an accelerated reproductive senescence. An accelerated senescence in gamete production in individuals investing more in sexual display has also been shown for male houbara bustards (Preston et al., 2011). Evolutionary trade‐offs between female mating system and longevity have been reported in a handful of studies. Female seed beetles (*Callosobruchus maculatus*) evolving under polygamy for 35 generations showed reduced life span compared to females evolving under enforced monogamy, although this difference was due to baseline rather that age‐specific mortality (Maklakov et al., 2007). Similarly, Travers et al. (2015) reported a negative genetic correlation between mating frequency and longevity in fruitflies (*Drosophila melanogaster*), indicating that females with a genetic propensity to mate multiply have shorter life span. Such trade‐offs might also contribute to explain the within‐population coexistence of monoandrous and polyandrous females. Although whether a female engage in multiple mating is a trait with a substantial environmental determinism, several studies have also reported evidence for additive genetic variation of polyandry (Reid, 2012; Sutter et al., 2019; Travers et al., 2016). This raises the question of the maintenance of such polymorphism, if polyandry incurs a net fitness benefit to females. Future work in the houbara bustard might further elucidate whether age‐associated adjustment of reproductive investment differs between females from monoandrous and polyandrous lines that have been released in the wild.

Our findings contribute to inform the management of reproductive strategies within captive populations of endangered species. In conservation breeding programs aiming to restore declining natural populations through translocation, females usually do not have the opportunity to express any mate choice, neither at the pre‐ nor at the postcopulatory level. While enforced monogamy might be the easiest strategy to implement, because it allows to equalize the genetic representation of founders by mating individuals with the lowest mean kinship, and to maintain genetic diversity, its consequences for the population fitness might be questionable, and especially so for species that do not have a monogamous mating system in nature (Chargé et al., 2014). In agreement with this, recent work has shown that at least in some endangered species, allowing individuals to breed with a preferred mate (precopulatory sexual selection) improves reproductive success in captivity (Martin‐Wintle et al., 2015; Parrott et al., 2019). Our results show that allowing postcopulatory selection to operate also improves several components of female reproductive success. Therefore, full removal of sexual selection (both pre‐ and postcopulatory) might hinder population fitness of endangered species maintained in ex situ conservation programs. We further suggest that the consequences of removing sexual selection might extend to the individuals released in the wild to restore declining natural populations, as descendants of polyandrous females might be better able to found viable populations under more adverse environmental conditions (Power, & Holman, 2014). That said, allowing individuals to choose a mate is not feasible in all species maintained in captive breeding programs, because of among‐individual aggressive interactions, or the limited number of available individuals. In addition to this, even in species where male reproductive success is highly skewed (suggesting that sexual selection can operate), identifying the traits underlying female preference might be a difficult task, preventing the implementation of mate choice in the management of the captive populations (Farquharson et al., 2020). Finally, any benefit of sexual selection in terms of population fitness should be weighed against the risk of depauperating genetic diversity, if only a few individuals contribute to the next generation (Gooley et al., 2018). With this respect, it might be particularly useful to identify whether the beneficial effects arise from directional (e.g., selection for good genes) or nondirectional (e.g., selection for compatible genes) sexual selection. If population fitness increases as a consequence of directional selection consistently favoring few males, then we might expect that short‐term benefits might come at the expense of long‐term loss of genetic diversity. However, if improved population fitness results from the increased probability to match compatible partners, then implementing a strategy where females are allowed to “make a choice” might not necessarily produce a long‐term reduction in genetic diversity.

## CONFLICT OF INTEREST

The authors declare no conflict of interest.

## Supporting information

Supplementary Material

## Data Availability

The data that support the findings of this study are openly available in DRYAD at https://doi.org/10.5061/dryad.f4qrfj6wz.

## References

[eva13311-bib-0001] Arbuthnott, D. , & Rundle, H. D. (2012). Sexual selection is ineffectual or inhibits the purging of deleterious mutations in *Drosophila melanogaster* . Evolution, 66, 2127–2137. 10.1111/j.1558-5646.2012.01584.x 22759290

[eva13311-bib-0002] Ashley, M. V. , Willson, M. F. , Pergams, O. R. W. , O’Dowd, D. J. , Gende, S. M. , & Brown, J. S. (2003). Evolutionarily enlightened management. Biological Conservation, 111, 115–123. 10.1016/S0006-3207(02)00279-3

[eva13311-bib-0003] Barbosa, M. , Connolly, S. R. , Hisano, M. , Dornelas, M. , & Magurran, A. E. (2012). Fitness consequences of female multiple mating: A direct test of indirect benefits. BMC Evolutionary Biology, 12, 185. 10.1186/1471-2148-12-185 22978442 PMC3499236

[eva13311-bib-0004] Birkhead, T. R. , & Pizzari, T. (2002). Postcopulatory sexual selection. Nature Reviews Genetics, 3, 262–273. 10.1038/nrg774 11967551

[eva13311-bib-0005] Boulton, R. A. , Zuk, M. , & Shuker, D. M. (2018). An inconvenient truth: The unconsidered benefits of convenience polyandry. Trends in Ecology and Evolution, 33, 904–915. 10.1016/j.tree.2018.10.002 30376988

[eva13311-bib-0006] Brouwer, L. , & Griffith, S. C. (2019). Extra‐pair paternity in birds. Molecular Ecology, 28, 4864–4882. 10.1111/mec.15259 31587397 PMC6899757

[eva13311-bib-0007] Cally, J. G. , Stuart‐Fox, D. , & Holman, L. (2019). Meta‐analytic evidence that sexual selection improves population fitness. Nature Communications, 10, 2017. 10.1038/s41467-019-10074-7 PMC6494874 31043615

[eva13311-bib-0008] Chapman, T. , Liddle, L. F. , Kalb, J. M. , Wolfner, M. F. , & Partridge, L. (1995). Cost of mating in *Drosophila melanogaster* females is mediated by male accessory gland products. Nature, 373, 241–244. 10.1038/373241a0 7816137

[eva13311-bib-0009] Chargé, R. , Teplitsky, C. , Hingrat, Y. , Saint Jalme, M. , Lacroix, F. , & Sorci, G. (2013). Quantitative genetics of sexual display, ejaculate quality and size in a lekking species. Journal of Animal Ecology, 82, 399–407. 10.1111/1365-2656.12023 23228188

[eva13311-bib-0010] Chargé, R. , Teplitsky, C. , Sorci, G. , & Low, M. (2014). Can sexual selection theory inform genetic management of captive populations? A review. Evolutionary Applications, 7, 1120–1133. 10.1111/eva.12229 25553072 PMC4231600

[eva13311-bib-0011] Chenoweth, S. F. , Appleton, N. C. , Allen, S. L. , & Rundle, H. D. (2015). Genomic evidence that sexual selection impedes adaptation to a novel environment. Current Biology, 25, 1860–1866. 10.1016/j.cub.2015.05.034 26119752

[eva13311-bib-0012] Croshaw, D. A. , Pechmann, J. H. K. , Glenn, T. C. , & Hebets, E. (2017). Multiple paternity benefits female marbled salamanders by increasing survival of progeny to metamorphosis. Ethology, 123, 307–315. 10.1111/eth.12597

[eva13311-bib-0013] Crudgington, H. S. , & Siva‐Jothy, M. T. (2000). Genital damage, kicking and early death – The battle of the sexes takes a sinister turn in the bean weevil. Nature, 407, 855–856. 10.1038/35038154 11057654

[eva13311-bib-0014] Demont, M. , Grazer, V. M. , Michalczyk, L. , Millard, A. L. , Sbilordo, S. H. , Emerson, B. C. , Gage, M. J. G. , & Martin, O. Y. (2014). Experimental removal of sexual selection reveals adaptations to polyandry in both sexes. Evolutionary Biology, 41, 62–70. 10.1007/s11692-013-9246-3

[eva13311-bib-0015] Ebenhard, T. (1995). Conservation breeding as a tool for saving animal species from extinction. Trends in Ecology and Evolution, 10, 438–443. 10.1016/S0169-5347(00)89176-4 21237098

[eva13311-bib-0016] Edward, D. A. , Fricke, C. , & Chapman, T. (2010). Adaptations to sexual selection and sexual conflict: Insights from experimental evolution and artificial selection. Philosophical Transactions of the Royal Society B, 365, 2541–2548. 10.1098/rstb.2010.0027 PMC2935098 20643744

[eva13311-bib-0017] Evans, J. P. (2012). Lifetime number of mates interacts with female age to determine reproductive success in female guppies. PLoS One, 7, e47507. 10.1371/journal.pone.0047507 23071816 PMC3470546

[eva13311-bib-0018] Evans, J. P. , & Magurran, A. E. (2000). Multiple benefits of multiple mating in guppies. Proceedings of the National Academy of Sciences USA, 97, 10074–10076. 10.1073/pnas.180207297 PMC27698 10954750

[eva13311-bib-0019] Farquharson, K. A. , Hogg, C. J. , Belov, K. , & Grueber, C. E. (2020). Deciphering genetic mate choice: Not so simple in group‐housed conservation breeding programs. Evolutionary Applications, 13, 2179–2189. 10.1111/eva.12981 33005217 PMC7513713

[eva13311-bib-0020] Farquharson, K. A. , Hogg, C. J. , & Grueber, C. E. (2019). A case for genetic parentage assignment in captive group housing. Conservation Genetics, 20, 1187–1193. 10.1007/s10592-019-01198-w

[eva13311-bib-0021] Firman, R. C. , & Simmons, L. W. (2012). Male house mice evolving with post‐copulatory sexual selection sire embryos with increasing viability. Ecology Letters, 15, 42–46. 22011211 10.1111/j.1461-0248.2011.01706.x

[eva13311-bib-0022] Fisher, D. O. , Double, M. C. , Blomberg, S. P. , Jennions, M. D. , & Cockburn, A. (2006). Post‐mating sexual selection increases lifetime fitness in polyandrous females. Nature, 444, 89–92. 17080089 10.1038/nature05206

[eva13311-bib-0023] Fitzpatrick, J. L. , & Lüpold, S. (2014). Sexual selection and the evolution of sperm quality. Molecular Human Reproduction, 20, 1180–1189. 10.1093/molehr/gau067 25323970

[eva13311-bib-0024] Forstmeier, W. , Nakagawa, S. , Griffith, S. C. , & Kempenaers, B. (2014). Female extra‐pair mating: Adaptation or genetic constraint? Trends in Ecology and Evolution, 29, 456–464. 10.1016/j.tree.2014.05.005 24909948

[eva13311-bib-0025] Gasparini, C. , & Evans, J. P. (2018). Female control over multiple matings increases the opportunity for postcopulatory sexual selection. Proceedings of the Royal Society B, 285, 20181505. 10.1098/rspb.2018.1505 30282652 PMC6191706

[eva13311-bib-0026] Gerlach, N. M. , McGlothlin, J. W. , Parker, P. G. , & Ketterson, E. D. (2012). Promiscuous mating produces offspring with higher lifetime fitness. Proceedings of the Royal Society B, 279, 860–866. 10.1098/rspb.2011.1547 21881136 PMC3259935

[eva13311-bib-0027] Gilbert, L. , Williamson, K. A. , & Graves, J. A. (2012). Male attractiveness regulates daughter fecundity non‐genetically via maternal investment. Proceedings of the Royal Society B, 279, 523–528. 10.1098/rspb.2011.0962 21733898 PMC3234556

[eva13311-bib-0028] Godwin, J. L. , Lumley, A. J. , Michalczyk, L. , Martin, O. Y. , & Gage, M. J. G. (2020). Mating patterns influence vulnerability to the extinction vortex. Global Change Biology, 26, 4226–4239. 10.1111/gcb.15186 32558066

[eva13311-bib-0029] Gooley, R. M. , Hogg, C. J. , Belov, K. , & Grueber, C. E. (2018). The effect of group versus intensive housing on the retention of genetic diversity in insurance populations. BMC Zoology, 3, 2.

[eva13311-bib-0030] Gowaty, P. A. , Kim, Y.‐K. , Rawlings, J. , & Anderson, W. W. (2010). Polyandry increases offspring viability and mother productivity but does not decrease mother survival in *Drosophila pseudoobscura* . Proceedings of the National Academy of Sciences USA, 107, 13771–13776. 10.1073/pnas.1006174107 PMC2922267 20643932

[eva13311-bib-0031] Head, M. L. , Hunt, J. , Jennions, M. D. , & Brooks, R. (2005). The indirect benefits of mating with attractive males outweigh the direct costs. PLoS Biology, 3, e33. 10.1371/journal.pbio.0030033 15678167 PMC544928

[eva13311-bib-0032] Hingrat, Y. , Saint Jalme, M. , Ysnel, F. , Le Nuz, E. , & Lacroix, F. (2007). Habitat use and mating system of the houbara bustard (*Chlamydotis undulata undulata*) in a semi‐desertic area of North Africa: Implications for conservation. Journal of Ornithology, 148, 39–52. 10.1007/s10336-006-0098-9

[eva13311-bib-0033] Holland, B. (2002). Sexual selection fails to promote adaptation to a new environment. Evolution, 56, 721–730. 10.1111/j.0014-3820.2002.tb01383.x 12038530

[eva13311-bib-0034] Holman, L. (2015). Bet‐hedging via multiple mating: A meta‐analysis. Evolution, 70, 62–71. 10.1111/evo.12822 26596684

[eva13311-bib-0035] Holman, L. , & Kokko, H. (2013). The consequences of polyandry for population viability, extinction risk and conservation. Philosophical Transactions of the Royal Society B, 368, 20120053. 10.1098/rstb.2012.0053 PMC3576587 23339244

[eva13311-bib-0036] Kokko, H. , & Mappes, J. (2012). Multiple mating by females is a natural outcome of a null model of mate encounters. Entomologia Experimentalis Et Applicata, 146, 26–37.

[eva13311-bib-0037] Lesobre, L. , Lacroix, F. , Caizergues, A. , Hingrat, Y. , Chalah, T. , & Saint Jalme, M. (2010). Conservation genetics of houbara bustard (*Chlamydotis undulata undulata*): Population structure and its implications for the reinforcement of wild populations. Conservation Genetics, 11, 1489–1497. 10.1007/s10592-009-9979-9

[eva13311-bib-0038] Lesobre, L. , Lacroix, F. , Le Nuz, E. , Hingrat, Y. , Chalah, T. , & Saint Jalme, M. (2010). Absence of male reproductive skew, along with high frequency of polyandry and conspecific brood parasitism in the lekking houbara bustard *Chlamydotis undulata undulata* . Journal of Avian Biology, 41, 117–127.

[eva13311-bib-0039] Lew, T. A. , Morrow, E. H. , & Rice, W. R. (2006). Standing genetic variance for female resistance to harm from males and its relationship to intralocus sexual conflict. Evolution, 60, 97–105. 10.1111/j.0014-3820.2006.tb01085.x 16568635

[eva13311-bib-0040] Lumley, A. J. , Diamond, S. E. , Einum, S. , Yeates, S. E. , Peruffo, D. , Emerson, B. C. , & Gage, M. J. G. (2016). Post‐copulatory opportunities for sperm competition and cryptic female choice provide no offspring fitness benefits in externally fertilizing salmon. Royal Society Open Science, 3, 150709. 10.1098/rsos.150709 27069665 PMC4821276

[eva13311-bib-0041] Lumley, A. J. , Michalczyk, L. , Kitson, J. J. N. , Spurgin, L. G. , Morrison, C. A. , Godwin, J. L. , Dickinson, M. E. , Martin, O. Y. , Emerson, B. C. , Chapman, T. , & Gage, M. J. G. (2015). Sexual selection protects against extinction. Nature, 522, 470–473. 10.1038/nature14419 25985178

[eva13311-bib-0042] Maklakov, A. A. , Fricke, C. , & Arnqvist, G. (2007). Sexual selection affects lifespan and aging in the seed beetle. Aging Cell, 6, 739–744. 10.1111/j.1474-9726.2007.00333.x 17725688

[eva13311-bib-0043] Martin, O. Y. , Hosken, D. J. , & Ward, P. I. (2004). Post‐copulatory sexual selection and female fitness in *Scatophaga stercoraria* . Proceedings of the Royal Society B, 271, 353–359. 15101693 10.1098/rspb.2003.2588 PMC1691601

[eva13311-bib-0044] Martinez‐Ruiz, C. , & Knell, R. J. (2017). Sexual selection can both increase and decrease extinction probability: Reconciling demographic and evolutionary factors. Journal of Animal Ecology, 86, 117–127. 10.1111/1365-2656.12601 27861841

[eva13311-bib-0045] Martin‐Wintle, M. S. , Shepherdson, D. , Zhang, G. , Zhang, H. , Li, D. , Zhou, X. , Li, R. , & Swaisgoog, R. R. (2015). Free mate choice enhances conservation breeding in the endangered giant panda. Nature Communications, 6, 10125. 10.1038/ncomms10125 PMC4682106 26670381

[eva13311-bib-0046] Martin‐Wintle, M. S. , Wintle, N. J. P. , Diez‐Leon, M. , Swaisgood, R. R. , & Asa, C. S. (2018). Improving the sustainability of ex situ populations with mate choice. Zoo Biology, 2018, 1–14. 10.1002/zoo.21450 30474268

[eva13311-bib-0047] Nandy, B. , Chakraborty, P. , Gupta, V. , Ali, S. Z. , & Prasad, N. G. (2013). Sperm competitive ability evolves in response to experimental alteration of operational sex ratio. Evolution, 67, 2133–2141. 10.1111/evo.12076 23815666

[eva13311-bib-0048] Newcomer, S. D. , Zeh, J. A. , & Zeh, D. W. (1999). Genetic benefits enhance the reproductive success of polyandrous females. Proceedings of the National Academy of Sciences USA, 96, 10236–10241. 10.1073/pnas.96.18.10236 PMC17872 10468592

[eva13311-bib-0049] Parker, G. A. (2006). Sexual conflict over mating and fertilization: an overview. Philosophical Transactions of the Royal Society B, 361, 235–259. 10.1098/rstb.2005.1785 PMC1569603 16612884

[eva13311-bib-0050] Parker, G. A. , & Pizzari, T. (2010). Sperm competition and ejaculate economics. Biological Reviews, 85, 897–934. 10.1111/j.1469-185X.2010.00140.x 20560928

[eva13311-bib-0051] Parrett, J. M. , Mann, D. J. , Chung, A. Y. C. , Slade, E. M. , & Knell, R. J. (2019). Sexual selection predicts the persistence of populations within altered environments. Ecology Letters, 22, 1629–1637. 10.1111/ele.13358 31353816

[eva13311-bib-0052] Parrott, M. L. , Nation, A. , & Selwood, L. (2019). Female mate choice significantly increases captive breeding success, and scents can be frozen to determine choice, in the stripe‐faced dunnart. Applied Animal Behaviour Science, 214, 95–101. 10.1016/j.applanim.2019.03.006

[eva13311-bib-0053] Pélabon, C. , Larsen, L. K. , Bolstad, G. H. , Viken, A. , Fleming, I. A. , & Rosenqvist, G. (2014). The effects of sexual selection on life history traits: An experimental study on guppies. Journal of Evolutionary Biology, 27, 404–416. 10.1111/jeb.12309 24417444

[eva13311-bib-0054] Pischedda, A. , & Chippindale, A. K. (2006). Intralocus sexual conflict diminishes the benefits of sexual selection. PLoS Biology, 4, 2099–2103. 10.1371/journal.pbio.0040356 PMC1618422 17105343

[eva13311-bib-0055] Power, D. J. , & Holman, L. (2014). Polyandrous female found fitter populations. Journal of Evolutionary Biology, 27, 1948–1955. 25039698 10.1111/jeb.12448

[eva13311-bib-0056] Preston, B. T. , Saint Jalme, M. , Hingrat, Y. , Lacroix, F. , & Sorci, G. (2011). Sexually extravagant males age more rapidly. Ecology Letters, 14, 1017–1024. 10.1111/j.1461-0248.2011.01668.x 21806745

[eva13311-bib-0057] Price, T. A. R. , Hurst, G. D. D. , & Wedell, N. (2010). Polyandry prevents extinction. Current Biology, 20, 471–475. 10.1016/j.cub.2010.01.050 20188561

[eva13311-bib-0058] Rabier, R. , Robert, A. , Lacroix, F. , & Lesobre, L. (2020). Genetic assessment of a conservation breeding program of the Houbara bustard (*Chlamydotis undulata undulata*) in Morocco, based on pedigree and molecular analyses. Zoo Biology, 39, 422–435. 32956518 10.1002/zoo.21569

[eva13311-bib-0059] Reid, J. M. (2012). Predicting evolutionary responses to selection on polyandry in the wild: Additive genetic covariances with female extra‐pair reproduction. Proceedings of the Royal Society B, 279, 4652–4660. 10.1098/rspb.2012.1835 22993252 PMC3479734

[eva13311-bib-0060] Saint Jalme, M. , Gaucher, P. , & Paillat, P. (1994). Artificial insemination in Houbara bustards (*Chlamydotis undulata*): influence of the number of spermatozoa and insemination frequency on fertility and ability to hatch. Journal of Reproduction and Fertility, 100, 93–103. 10.1530/jrf.0.1000093 8182618

[eva13311-bib-0061] Schulte‐Hostedde, A. I. , & Mastromonaco, G. F. (2015). Integrating evolution in the management of captive zoo populations. Evolutionary Applications, 8, 413–422. 10.1111/eva.12258 26029256 PMC4430766

[eva13311-bib-0062] Simmons, L. W. (2005). The evolution of polyandry: Sperm competition, sperm selection, and offspring viability. Annual Reviews in Ecology Evolution and Systematics, 36, 125–146. 10.1146/annurev.ecolsys.36.102403.112501

[eva13311-bib-0063] Simmons, L. W. , & Fitzpatrick, J. L. (2012). Sperm wars and the evolution of male fertility. Reproduction, 144, 519–534. 10.1530/REP-12-0285 22984191

[eva13311-bib-0064] Slatyer, R. A. , Mautz, B. S. , Blackwell, P. R. Y. , & Jennions, M. D. (2012). Estimating genetic benefits of polyandry from experimental studies: A meta‐analysis. Biological Reviews, 87, 1–33. 10.1111/j.1469-185X.2011.00182.x 21545390

[eva13311-bib-0065] Sutter, A. , Travers, L. M. , Weedon, M. , Oku, K. , Price, T. A. R. , & Wedell, N. (2019). No selection for change in polyandry under experimental evolution. Journal of Evolutionary Biology, 32, 717–730. 10.1111/jeb.13476 30970158

[eva13311-bib-0066] Taylor, M. L. , Price, T. A. R. , & Wedell, N. (2014). Polyandry in nature: A global analysis. Trends in Ecology and Evolution, 29, 376–383. 10.1016/j.tree.2014.04.005 24831458

[eva13311-bib-0067] Taylor, M. L. , Wigmore, C. , Hodgson, D. J. , Wedell, N. , & Hosken, D. J. (2008). Multiple mating increases female fitness in *Drosophila simulans* . Animal Behaviour, 76, 963–970. 10.1016/j.anbehav.2008.05.015

[eva13311-bib-0068] Thonhauser, K. E. , Raveh, S. , Hettyey, A. , Beissmann, H. , & Penn, D. J. (2013). Why do female mice mate with multiple males? Behavioral Ecology and Sociobiology, 67, 1961–1970. 10.1007/s00265-013-1604-8 24273373 PMC3827896

[eva13311-bib-0069] Travers, L. M. , Garcia‐Gonzalez, F. , & Simmons, L. W. (2015). Live fast fie young life history in females: evolutionary trade‐off between early life mating and lifespan in female *Drosophila melanogaster* . Scientific Reports, 5, 15469. 26482533 10.1038/srep15469 PMC4612512

[eva13311-bib-0070] Travers, L. M. , Simmons, L. W. , & Garcia‐Gonzalez, F. (2016). Additive genetic variance in polyandry enables its evolution, but polyandry is unlikely to evolve through sexy or good sperm processes. Journal of Evolutionary Biology, 29, 916–928. 10.1111/jeb.12834 26801640

[eva13311-bib-0071] Vuarin, P. , Bouchard, A. , Lesobre, L. , Levêque, G. , Chalah, T. , Saint Jalme, M. , Hingrat, Y. , Lacroix, F. , & Sorci, G. (2019). Post‐copulatory sexual selection allows females to alleviate the fitness costs incurred when mating with senescing males. Proceedings of the Royal Society B, 286, 20191675. 10.1098/rspb.2019.1675 31640511 PMC6834038

[eva13311-bib-0072] Vuarin, P. , Hingrat, Y. , Lesobre, L. , Saint Jalme, M. , Lacroix, F. , & Sorci, G. (2019). Sperm competition accentuates selection on ejaculate attributes. Biology Letters, 15, 20180889. 10.1098/rsbl.2018.0889 30890070 PMC6451384

[eva13311-bib-0073] Wedekind, C. (2002). Sexual selection and life‐history decisions: implications for supportive breeding and the management of captive populations. Conservation Biology, 16, 1204–1211. 10.1046/j.1523-1739.2002.01217.x

[eva13311-bib-0075] Wigby, S. , & Chapman, T. (2005). Sex peptide causes mating costs in female *Drosophila melanogaster* . Current Biology, 15, 316–321. 10.1016/j.cub.2005.01.051 15723791

