# Supplementary material for: Enforced monoandry over generations induces a reduction of female investment into reproduction in a promiscuous bird
Source: Evol Appl. 2021 Nov 13;14(12):2773–83. doi: 10.1111/eva.13311 (PMC8674888; doi:10.1111/eva.13311)
Supplement: Supplementary file 1 — Supplementary Material [file EVA-14-2773-s001.pdf]

Enforced monoandry over generations induces a reduction of female investment into reproduction in a promiscuous bird

Supplementary Information

Supplementary tables

Table S1. Linear mixed effects model exploring the effect of the mating system on date of first egg laid. The model included the mating system (monoandry or polyandry), age (log-transformed), squared age, and the two-way interactions between mating system and age. Line, year of birth and year of data collection were included as random effects. The analysis was based on 421 observations collected over 13 years on 87 female lines spanning 13 cohorts. The monoandrous group was set as the reference. We report parameter estimates (with SE and 95% CI), t and p values for the initial model.

**Initial model**					
Fixed effects	*Parameter estimate*	*SE*	*t*	*p*	*95% CI*
Intercept (monoandry)	84.04	3.95			
Mating system (polyandry)	1.40	3.77	0.37	0.7117	-6.03/8.82
Age	-3.10	3.55	-0.87	0.3833	-10.09/3.89
Age^2	-2.09	3.66	-0.57	0.5677	-9.28/5.10
Mating system (polyandry) x age	-7.04	5.88	-1.20	0.2318	-18.61/4.52
Mating system (polyandry) x age^2	7.30	6.36	1.15	0.2520	-5.22/19.82
Random effects	*Estimate*	*SE*	*z*	*p*	
Line	195.54	42.78	4.57	<0.0001	
Year of birth	91.46	69.18	1.32	0.0931	
Year of data collection	16.65	12.85	1.30	0.0975	
Residual	181.41	14.51	12.50	<0.0001	

Table S2. Linear mixed effects model exploring the effect of the mating system on the number of eggs laid per season. The model (which had a Poisson distribution of errors) included the mating system (monoandry or polyandry), age (log-transformed), squared age, maximum number of inseminations, maximum number of males, date of first egg laid, and the two-way interactions between mating system and covariates. Line, year of birth and year of data collection were included as random effects. The analysis was based on 609 observations collected over 13 years on 87 female lines spanning 13 cohorts. The monoandrous group was set as the reference. We report parameter estimates (with SE and 95% CI), t and p values for the initial model.

**Initial model**					
Fixed effects	*Parameter estimate*	*SE*	*t*	*p*	*95% CI*
Intercept (monoandry)	1.51	0.05			
Mating system (polyandry)	0.10	0.05	1.88	0.0603	-0.004/0.21
Age	0.39	0.11	3.59	0.0004	0.18/0.60
Age²	-0.40	0.10	-4.08	<0.0001	-0.60/-0.21
Maximum number of inseminations	0.45	0.03	13.31	<0.0001	0.38/0.51
Maximum number of males	0.19	0.03	6.95	<0.0001	0.14/0.24
Date of first egg laid	-0.16	0.02	-6.74	<0.0001	-0.21/-0.11
Mating system (polyandry) x age	-0.35	0.18	-1.99	0.0469	-0.69/-0.005
Mating system (polyandry) x age²	0.26	0.17	1.52	0.1294	-0.08/0.60
Mating system (polyandry) x maximum number of inseminations	0.03	0.06	0.43	0.6638	-0.09/0.14
Mating system (polyandry) x maximum number of males	0.02	0.05	0.32	0.7509	-0.08/0.11
Mating system (polyandry) x date of first egg laid	-0.007	0.04	-0.15	0.8812	-0.09/0.08
Random effects	*Estimate*	*SE*			
Line	0.009	0.006			
Year of birth	0.015	0.013			

Table S3. Linear mixed effects model exploring the effect of the mating system on egg mass (g). The model included the mating system (monoandry or polyandry), age (log-transformed), squared age, the egg position in the laying sequence, the number of males that contributed to the inseminations, laying date (linear and quadratic) and the two-way interactions between mating system and the covariates. Line, year of birth and year of data collection were included as random effects. The analysis was based on 1304 observations collected over 11 years on 74 female lines spanning 12 cohorts. The monoandrous group was set as the reference. We report parameter estimates (with SE and 95% CI), t and p values for the initial model.

**Initial model**					
Fixed effects	*Parameter estimate*	*SE*	*t*	*p*	*95% CI*
Intercept (monoandry)	62.02	1.06			
Mating system (polyandry)	1.97	1.38	1.43	0.1542	-0.74/4.67
Age	5.06	0.61	8.32	<0.0001	3.87/6.25
Age^2	-3.08	0.73	-4.20	<0.0001	-4.52/-1.64
Egg position	-1.49	0.26	-5.71	<0.0001	-2.01/-0.98
Number of males	-0.14	0.17	-0.87	0.3822	-0.47/0.18
Laying date	-0.08	0.60	-0.14	0.8895	-1.26/1.09
Laying $date^2$	0.83	0.61	1.37	0.1711	-0.36/2.03
Mating system (polyandry) x age	0.42	0.95	0.45	0.6549	-1.43/2.28
Mating system (polyandry) x age^2	-2.15	0.98	-2.19	0.0291	-4.08/-0.22
Mating system (polyandry) x egg position	0.74	0.50	1.50	0.1342	-0.23/1.72
Mating system (polyandry) x number of males	-0.16	0.29	-0.56	0.5761	-0.72/0.40
Mating system (polyandry) x laying date	-3.43	1.40	-2.45	0.0142	-6.17/-0.69
Mating system (polyandry) x laying $date^2$	1.78	1.29	1.37	0.1696	-0.76/4.32
Random effects	*Estimate*	*SE*	*z*	*p*	
Line	29.51	5.36	5.51	<0.0001	
Year of birth	0				
Year of data collection	3.66	1.86	1.97	0.0245	
Residual	9.93	0.40	24.56	<0.0001	

Table S4. Linear mixed effects model exploring the effect of the mating system on hatchling mass (g). The model included the mating system (monoandry or polyandry), age (log-transformed), squared age, the egg position in the laying sequence, the number of males that contributed to the inseminations, laying date (linear and quadratic) and the two-way interactions between mating system and the covariates. Line, year of birth and year of data collection were included as random effects. The analysis was based on 2718 observations collected over 13 years on 86 female lines spanning 13 cohorts. The monoandrous group was set as the reference. We report parameter estimates (with SE and 95% CI), t and p values for the initial model.

**Initial model**					
Fixed effects	*Parameter estimate*	*SE*	*t*	*p*	*95% CI*
Intercept (monoandry)	40.75	0.62			
Mating system (polyandry)	1.10	0.81	1.35	0.1756	-0.49/2.70
Age	3.15	0.33	9.55	<0.0001	2.50/3.79
Age^2	-1.98	0.39	-5.07	<0.0001	-2.75/-1.21
Egg position	-1.13	0.13	-9.00	<0.0001	-1.38/-0.88
Number of males	-0.24	0.08	-2.86	0.0042	-0.41/-0.08
Laying date	-0.78	0.32	-2.43	0.0151	-1.40/-0.15
Laying $date^2$	1.17	0.33	3.54	0.0004	0.52/1.81
Mating system (polyandry) x age	1.46	0.51	2.84	0.0046	0.45/2.46
Mating system (polyandry) x age^2	-2.48	0.55	-4.51	<0.0001	-3.55/-1.40
Mating system (polyandry) x egg position	-0.32	0.23	-1.36	0.1727	-0.78/0.14
Mating system (polyandry) x number of males	0.02	0.14	0.12	0.9051	-0.27/0.30
Mating system (polyandry) x laying date	-0.39	0.64	-0.61	0.5407	-1.65/0.87
Mating system (polyandry) x laying $date^2$	0.19	0.62	0.30	0.7636	-1.04/1.41
Random effects	*Estimate*	*SE*	*z*	*p*	
Line	12.58	2.05	6.14	<0.0001	
Year of birth	0				
Year of data collection	1.27	0.65	1.95	0.0258	
Residual	6.69	0.19	36.09	<0.0001	

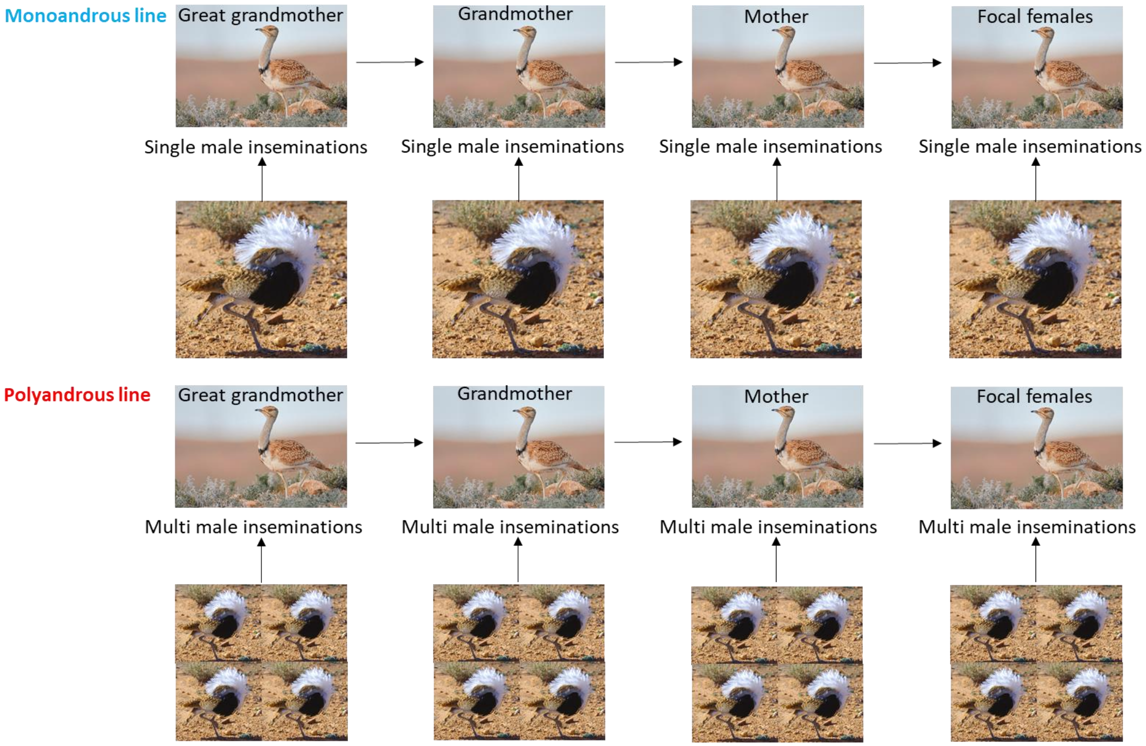

Figure S1. Schematic illustration of the rationale used to reconstruct the monoadrous and polyandrous lines. We defined as a monoandrous line a female that was produced following a single male insemination of her mother, whose mother was produced following a single male insemination of her grandmother, whose grandmother was produced following a single male insemination of her great grandmother. A polyandrous line was defined as a female that was produced following a multi male insemination of her mother, whose mother was produced following a multi male insemination of her grandmother, whose grandmother was produced following a multi male insemination of her great grandmother. We considered an individual to be eligible to be included in a monoandrous line only when the egg from which it hatched was laid following inseminations with sperm collected from single male during the 40 days that preceded laying. Note that the picture of four males in the polyandrous line is for illustrative purposes only, since the average number of males whose sperm was used in the polyandrous lines was 3.4 (min = 2, max = 8). We could only reconstruct female lines, the sample size of male lines produced following constant monoandrous or polyandrous inseminations being too small. Photo credit: Yves Hingrat.

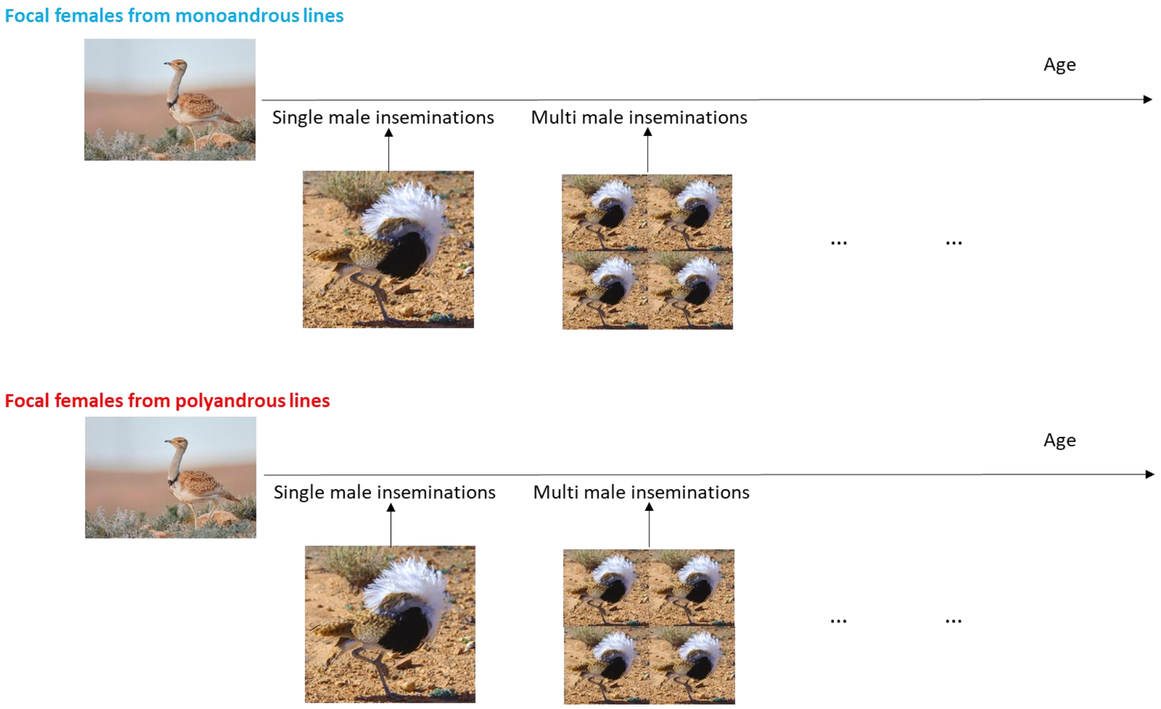

Figure S2. The third-generation females produced under a regime of constant monoandrous or polyandrous inseminations were used as focal individuals in this study. In particular, we investigated their reproductive traits (date of first egg laying, number of eggs laid, egg and hatchling mass) over their lifetime. Note that these females experienced both single male and multi male inseminations. The order of insemination type (as reported in the figure) is for illustrative purposes only, and does not indicate that females were first inseminated with single male sperm and then with multi male sperm. Photo credit: Yves Hingrat.

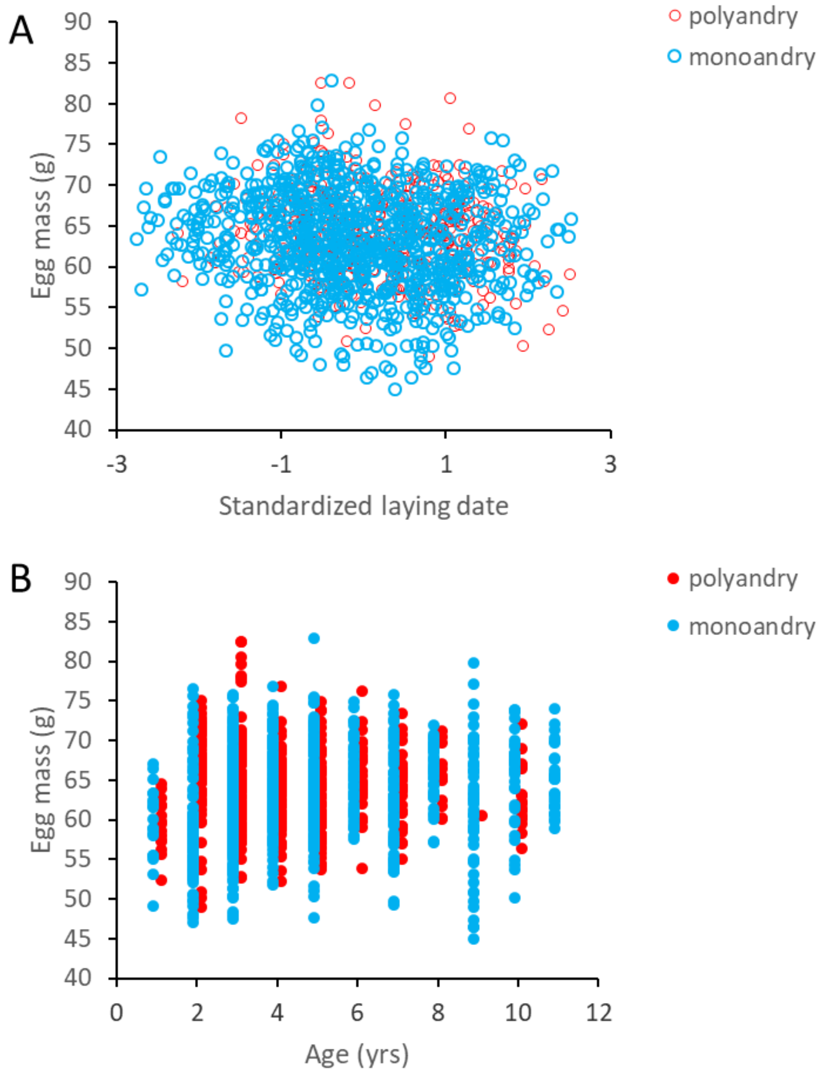

Figure S3. Egg mass (g) as a function of laying date (A) and female age (B) for the two mating systems (monoandry = blue dots; polyandry = red dots).

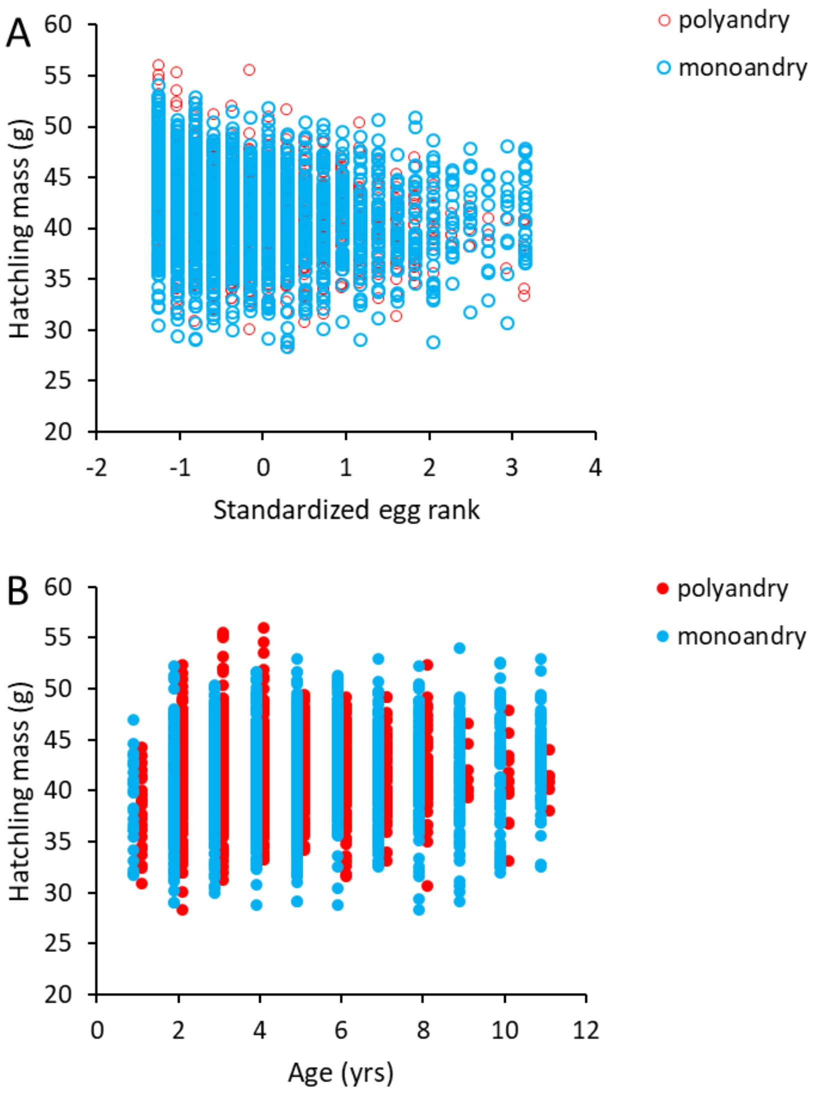

Figure S4. Hatchling mass (g) as a function of egg ranking in the laying sequence (A) and female age (B) for the two mating systems (monoandry = blue dots; polyandry = red dots).
